# Development of an Interactive Lifestyle Programme for Adolescents at Risk of Developing Type 2 Diabetes: PRE-STARt

**DOI:** 10.3390/children8020069

**Published:** 2021-01-21

**Authors:** Deirdre M. Harrington, Emer M. Brady, Susann Weihrauch-Bluher, Charlotte L. Edwardson, Laura J. Gray, Michelle Hadjiconstantinou, Janet Jarvis, Kamlesh Khunti, Itziar Vergara, Irati Erreguerena, Rogério T. Ribeiro, Jacqui Troughton, Andriani Vazeou, Melanie J. Davies

**Affiliations:** 1 Diabetes Research Centre, University of Leicester, Leicester LE5 4PW, UK; ce95@leicester.ac.uk (C.L.E.); mh333@leicester.ac.uk (M.H.); kk22@leicester.ac.uk (K.K.); 2 School of Psychological Sciences and Health, University of Strathclyde, Glasgow G1 1QE, UK; 3 Cardiovascular Sciences, University of Leicester, Leicester LE3 9QP, UK; emb29@leicester.ac.uk; 4 Leicester Diabetes Centre, University Hospitals of Leicester NHS Trust, Leicester LE5 4PW, UK; jj99@leicester.ac.uk (J.J.); Jacqui.Troughton@uhl-tr.nhs.uk (J.T.); melanie.davies@uhl-tr.nhs.uk (M.J.D.); 5 Integrated Research and Treatment Center (IFB) Adiposity Diseases, University of Leipzig, 04103 Leipzig, Germany; susann.weihrauch-blueher@uk-halle.de; 6 Department for Operative and Nonoperative Pediatric and Adolescent Medicine, University HospitalHalle (Saale), 06120 Halle (Saale), Germany; 7 Department of Health Sciences, University of Leicester, Leicester LE1 7RH, UK; lg48@leicester.ac.uk; 8 Instituto de Investigación en Servicios de Salud Kronikgune, 48902 Barakaldo (Bizkaia), Spain; MARIAICIAR.VERGARAMITXELTORENA@osakidetza.eus (I.V.); ierreguerena@kronikgune.org (I.E.); 9 Red de Investigación en Servicios de Salud en Enfermedades Crónicas-REDISSEC, 48902 Barakaldo (Bizkaia), Spain; 10 Education and Research Department (ERC), APDP-Portuguese Diabetes Association, 1250-189 Lisbon, Portugal; rogerio.ribeiro@apdp.pt; 11 Diabetes Center, Department of Pediatrics, P&A Kyriakou Children’s Hospital, 115 27 Athens, Greece; agerasim@gmail.com; 12 NIHR Leicester Biomedical Research Centre, Leicester LE5 4PW, UK

**Keywords:** behaviour change, prevention, physical activity, lifestyle, family

## Abstract

Background: Type 2 diabetes (T2D) is increasing in young people. Reporting on the processes used when developing prevention interventions is needed. We present the development of a family-based interactive lifestyle intervention for adolescents with risk factors for T2D in the future. Method: A multidisciplinary team in the UK site led the intervention development process with sites in Portugal, Greece, Germany and Spain. Potential programme topics and underpinning theory were gathered from literature and stakeholders. A theoretical framework based on self-efficacy theory and the COM-B (capability, opportunity, motivation, behaviour) model was developed. Sessions and supporting resources were developed and refined via two iterative cycles of session and resource piloting, feedback, reflection and refinement. Decision on delivery and content were made by stakeholders (young people, teachers, parents, paediatricians) and all sites. Materials were translated to local languages. Site-specific adaptations to the language, content and supporting resources were made. Results: The “PRE-STARt” programme is eight 90-min interactive sessions with supporting curriculum and resources. Iterative development work provided valuable feedback on programme content and delivery. Conclusion: Reporting on the intervention development process, which includes stakeholder input, could yield a flexible approach for use in this emerging ‘at risk’ groups and their families.

## 1. Introduction

Type 2 diabetes (T2D) is a serious chronic and progressive disease that leads to both microvascular and macrovascular complications [1]. Poor glycaemic control, the hallmark of T2D, is now estimated to be one of the top ten leading causes of death globally [2] and the annual healthcare burden is high [3,4]. Traditionally a condition of old age, T2D is increasingly being diagnosed in children, adolescents and young adults [5,6,7]. For example, US data projects a quadrupling of T2D for those under the age of 20 by 2050 [8]. 

T2D in younger people represents an extreme and aggressive phenotype that magnifies the disease profile observed in older adults and has multiple risk factors and comorbid conditions [9,10,11]. Obesity is a key biological driver for T2D (84.7% of children with T2D are obese [12]) and as tackling obesity continues to be a challenge [13] the increase in T2D in younger people continues to increase. Given the increasing prevalence and severity of T2D in younger populations and that more young people have established risk factors, early interventions to reduce the risk of developing T2D are increasingly called for, bringing together the clinical and community arenas [14,15,16]. 

T2D prevention can take place in multiple settings individually or acting together (i.e., school, community and family) but evidence shows that family involvement is essential in T2D prevention [17] and in healthy lifestyle programmes with schools and/or families [18,19,20]. This is unsurprising, given that the home environment exerts huge influence on children’s behaviours and overall health [21,22], especially for younger children. Furthermore, targeting multiple behaviours with direct family involvement can improve weight and related behaviours [19,21]. For example, the TODAY study (for young people with T2D) included a family-based behaviour change component that encouraged healthy eating, physical activity and weight loss [23]. 

Despite the number of interventions tested, there is scarce detailed knowledge and transparent reporting of the development process of intervention and programme development [24]. In particular, reporting according to consensus-based guidelines [25] is needed to improve scientific rigour and facilitate learning between peers and across disciplines [24]. To maximise the relevance and increase potential for success, programmes developed using stakeholder engagement and input are particularly valuable [26].

The aim of this paper is to describe the development process of an interactive lifestyle programme for adolescents aged 12–14 with risk factors which may increase their chances of developing T2D and their families (the ‘PRE-STARt’ programme) that is suitable for delivery in five different European countries.

## 2. Materials and Methods

### 2.1. Design, Setting and Target Population

Between February 2014 and December 2017, five sites in Europe-Leicester, United Kingdom (UK); Leipzig, Germany; Lisbon, Portugal; Athens, Greece; and Basque Country, Spain-collaborated on a European Commission funded project on work originally titled as “pilot prevention strategies for adolescents at risk of diabetes”. Subsequent to the development of this programme, we developed a T2D risk identification tool that could help identify young people aged 12 to 14 who would potentially be at increased risk of developing T2D in the future and therefore benefit from the lifestyle programme [27]. Alongside that, the UK site led the development of the lifestyle programme in collaboration with the four other European sites. The prevailing framework in the UK for developing complex interventions (Medical Research Council guidance) [28] and the practical set of sequential steps in the Leicester Diabetes Centre pathway for intervention development [29] were followed. The activities supporting programme development are presented in chronological order in Table S1. These included engagement and meetings with stakeholders to make decisions on programme content and delivery. Our development process is reported herein, following the 14-item quality criteria from the recent GUIDED checklist and reporting guidance [25].

### 2.2. Working Group

A multidisciplinary development team (working group) of structured education specialists (*n* = 3), dietitian (*n* = 1) physical activity and sedentary behaviour researchers (*n* = 2), psychologists (*n* = 2) and healthcare professionals (*n* = 4) was established at the UK site. Teleconferences between all sites occurred when needed (typically monthly but frequency increased depending on need and stage of development) to share ideas and discuss site-specific and overall progress of the collaboration. This development team worked closely with each collaborating site to discuss the programme, gather feedback and make refinements as the work progressed. The guiding principles of this work were to share experiences, resources and models of education and care, and ultimately develop a programme that encompassed ‘the best’ from each collaborating site. The collective approach to working meant that a core vision and shared aims were developed and agreed upon. The UK team worked with the research teams and clinicians (mainly primary care paediatricians, pediatric endocrinologists and diabetologists) from each site via email, teleconference and in person (see Table S2 for details) in developing the draft programme. Site visits were undertaken by the UK team.

### 2.3. Development Process

Literature search: In order to form a base for intervention content and delivery, a scoping literature search explored the approach to T2D prevention in young people in previous work in the field up to 2015. Programmes related to T2D, obesity or healthy lifestyles in young people were searched to identify evidence-based approaches. Information on programme and session structure (e.g., number and length of sessions; presence of parents with children), content and reported key learnings were extracted from studies. Online searches for freely available content and materials was also undertaken. Theories and philosophies: In tandem, searches for theories and philosophies relevant to the target age group were undertaken to inform the programme. A number of underpinning theories, philosophies and concepts drawn from the fields of behaviour change psychology and education were reviewed to inform the style, content and delivery methods used. A shared delivery philosophy (as well as training) is crucial to allow for standardised delivery as much as possible across and within sites.

Stakeholders: Each site gathered key stakeholders whose input was used to inform the development of the programme. Stakeholders included young people, teachers, parents, paediatricians and other school or community staff who may be involved in the delivery or commissioning of health and lifestyle-related programmes in the health or education systems. Information from the literature search was used as a base for getting stakeholder input during the activities used to guide choices on programme content (see Table S1).

Delivery personnel: An agreed person specification was developed to account for facilitators from different professional backgrounds and experiences to deliver the PRE-STARt group sessions. This outlined the:

- Skills: facilitator, group management;
- Behaviours: non-judgemental, empathetic;
- Knowledge: trained in the curriculum philosophies and content, understand the principles of supporting healthy lifestyle changes, understand how peer support and self-efficacy are used.

The skills, behaviours and knowledge that facilitators would be required to have were developed. These aligned with the agreed shared philosophy of the curriculum. 

Decision on session topics and delivery format: The programme was proposed as a series of interactive group-based sessions following a curriculum and supported by a range of engaging resources. In-person workshop and curriculum development meetings (frequency differed depending on stage of development but ranged from weekly to monthly) began in the UK site as a forum where materials collated from similar programmes were presented and discussed. The collaborative agreed to put less emphasis on T2D and instead focus on promoting healthier lifestyle choices which positively influence risk reduction for many chronic conditions as per feedback from the Spanish site. 

A UK site stakeholder meeting used an eVoting system to help make key decisions on the programme content and delivery (see activity #7 in Table S1). Up to this point a list of 27 potential topics for the programme were gathered from the literature, what was topical in the media and from young people’s suggestions. At this meeting the attendees ranked these topics in order of importance. Decisions on session number and length, delivery timetable, parent/guardian involvement and resource use were also made. Each site decided on a delivery setting appropriate for their local context. 

Changing physical activity levels, in particular moderate-to-vigorous intensity physical activity (MVPA), was the main behavioural target of the programme. This was chosen ahead of a weight-related outcome due to the multiple physical and mental benefits a change in physical activity has that a change in weight does not. There was discussion on whether some physical activity should be included in each session or whether the young people could engage in physical activity while education was delivered to the parents. It was decided by the collaborative to include a physical activity session where the parent and child could be active together for 10 min at the end of each session. The activities were designed to be either co-operative, having parents switch children, or having all pairs co-operate. As well as giving the participant much needed physical activity, it would foster a sense of enjoyment from being active together and bonding. 

Self-monitoring: Self-monitoring and goal setting are key features of the PRE-STARt theoretical framework. Wearable technology was considered as an option to support this through real-time feedback on physical activity and sedentary time. Any device needed to be suitable for this age group, have feedback that aligns with the intervention content and goals, be acceptable to young people and minimise burden on facilitators who would be responsible for setting up the devices in the first session. A member of the research team (CE) reviewed the commercially available physical activity self-monitoring tools for under £100 and this helped guide the discussion. Features of the devices, including wear location, battery life, feedback, physical activity-related outputs (steps, distance, calories, duration, intensity and type) and cost, were presented to representatives from the collaborating sites. 

One commercially available activity tracker (the Polar LoopTM Activity Tracker, Polar, Kempele, Finland) was trialed with nine adolescents in the UK. A feedback session was undertaken with these adolescents, two parents and a teacher. They were positive about the device’s display and feedback screen, reported needing more assistance with initial device setup and they felt a modern wrist-worn device was a cooler alternative to the traditional waistband-worn pedometer. Despite positive remarks, an alternative was sought due to the technical needs of the initial setup which could add a burden for the facilitators. The Garmin VivoFit2TM (Garmin Ltd., Schaffhausen, Switzerland) was decided upon due to a balance between activity feedback, unit cost and simplicity to set up. 

Piloting and refinement of the draft sessions and resources: Young people involved in trying out the resources gave their verbal consent and their teacher gave written consent. As no data were collected from these young people, written consent was not needed from them or their parent/guardian. The study was conducted in accordance with the Declaration of Helsinki, and the overall protocol was approved by the NHS Research Ethics Service (NRES) Committee East Midlands–Leicester South Research Ethics Committee (16/EM/0221). Two iterative cycles (running each session, collecting feedback, reflection and modification of the sessions) were used to inform and refine the individual sessions and the overall programme. In cycle one, four testing and feedback sessions (5 to 11 young people aged 12 to 15 years; teachers present at all times) were undertaken in the UK site where the main constituent pieces of each session were described, the resources were tested and feedback was received from the attendees (Table S3). A separate session was conducted with 60 young people where the physical activities on the activity cards were tested. Observations, verbal feedback and discussions took place and themes noted by the research team. Feedback received across those testing sessions included: reduce wording on the worksheets; change images where meaning was not clear; have more time allocated to completing worksheets; increase the size of some group resources to ensure everyone can see; make imagery and food and drink examples more relatable for young people; and provide multiples of some resources so a large group can be split. 

Teachers’ feedback advised that the philosophy of supporting participants to discover and work out knowledge for themselves (i.e., that they are not passive receivers of information) as per the DESMOND programme for adults with T2D [30] would not be appropriate with younger groups. They advised that the Teacher Effectiveness Enhancement Programme (TEEP) model would allow facilitators to move away from trying to elicit knowledge from young people as, if they did not have lived experiences, it would take too much time and may cause disengagement. It is acceptable to provide new knowledge once the TEEP model was followed and then this new knowledge can be personalised and built upon in the session with parents. 

The second cycle involved a full running of each 90-min session over the school summer holidays at a school in a deprived multicultural area of the UK. A schoolteacher was trained to deliver the sessions. Structured written observations were taken by an Intervention Development Team member. Observations and verbal feedback were gathered following each workshop. Minor refinements to six sessions were identified, and incorporated, as was the need to provide additional detail in the facilitator manual to support facilitators. The content of sessions remained unchanged at this stage. 

### 2.4. Preparation for Delivery Across Sites

Details of underpinning programme theoretical and philosophical framework were added to a facilitator manual. To further support the facilitators, specific examples of “theory in action” were included in the manual. It was identified that facilitators would come from differing fields of education and/or healthcare, and a “person specification” of the typical knowledge, behaviour and skills that would be required of the facilitators was produced by the UK site and was used by each site to identify and train facilitators. The UK site coordinated the professional design of the facilitator manual, curriculum and participant materials. 

Over the course of four months, all documents were translated to each site’s local language. The design of the sessions allowed adjustment to each country’s specific nutrition, physical activity and health culture and messages. The main differences between sites were around nutrition and food items and references to eating patterns were adapted to reflect the diversity in how each site eats. Local versions of foods and health messages were used (e.g., the UK EatWell plate was replaced with an appropriate local equivalent at each site). These changes were made as they did not affect the underlying message, theory or philosophy. Regular telephone conferences between all participating sites (typically monthly but frequency was increased depending on need and development stage) and one on-site meetings at UK were organized to monitor the progress of the development process and to discuss next steps. 

## 3. Results

We present the results of the intervention development process. The Template for Intervention Description and Replication (TIDieR) checklist [31] was used to ensure high-quality reporting of the PRE-STARt programme (Table S4).

### 3.1. The Final Programme

The PRE-STARt programme comprises eight 90-min face-to-face group sessions. The programme can be delivered in person by one facilitator once they have been trained in the underpinning theories, as well as in delivery of the curriculum, facilitator manual and resources. Up to eight families can be accommodated per session (a family being one young person and one parent/guardian with additional parent/guardians and siblings welcome to attend ad hoc). 

Philosophy: The style of delivering PRE-STARt is underpinned by a number of philosophical beliefs (Table 1). Firstly, the family is ultimately responsible for the lifestyle choices the young person makes. Secondly, families want to maximise their quality of life and will make decisions accordingly. Thirdly, the family is best placed to identify any barriers to implementing new changes and identify any solutions to these. Each of these have implications for the delivery style and how the facilitator delivers the programme (see Table S3). 

Behaviour change theory, model content: Based on content of the extant literature, PRE-STARt aimed to elicit positive behaviour change through increased physical activity, reduced sedentary behaviour and healthier diet choices. The ability to get young people and the family to make a behaviour change is key, as well as delivering information and education. The learning theory used with the young people was guided by TEEP. To maximise the potential benefits for participants, the programme is underpinned by a number of theoretical concepts (see Table 1 for the theoretical framework). The facilitator manual contained practical examples of these theoretical concepts in action within the sessions.

Self-efficacy theory: Confidence in one’s ability to make a lifestyle change is a strong predictor of behaviour change. It was deemed important to develop a programme that would build confidence in children and their families to be able be more active, sit less and make healthy food choices. Self-efficacy theory embraces building confidence in one’s ability to make change and keep it going. This theory suggests there are four key strategies to enhance self-efficacy, and we ensured these were interlinked and embedded throughout each session. These strategies consisted of:

mastery experience (building confidence in small steps and allowing children to learn a new skill by attempting a new behaviour);
vicarious learning (encouraging children to share personal experiences and involving parents and educator to act out the desired behaviour);
verbal persuasion (eliciting a positive environment for children to believe that they are capable of adopting new behaviours and skills);
emotional states (allowing children to recognise physiological responses that might be experienced with the new behaviour).

To build confidence in attendees, a coaching approach was adopted by those facilitating the intervention. Sessions were delivered in a safe, empathetic and non-judgmental environment. Beliefs and values were shared and scaffolded in such a way that children were able to build on their skills and confidence (self-efficacy) throughout each session. 

**Figure 1:**
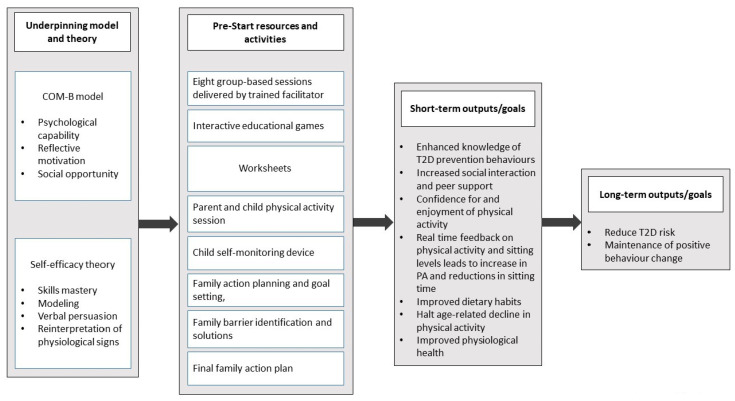
PRE-STARt logic model. This figure has been uploaded separately to improve quality.

COM-B model and the Theoretical Domains Framework: In addition to self-efficacy theory, we also wanted to consider a framework that specifically considered behaviour change (Table 2). We used the Theoretical Domains Framework (TDF) [32], which provides a theoretical basis for understanding behaviour change and simplifies 33 theories into 12 domains. We also mapped the COM-B (capability, opportunity, motivation behaviour) model [33], which distils the TDF into three key domains, proposing that people require capability, opportunity and motivation to perform a behaviour. For the purpose of our study, we mapped our intervention components into the components of the TDF and COM-B model (see Table 2) and developed a logic model (Figure 1), and we presented the “active ingredients” of our intervention by mapping a set of behaviour change techniques onto the TDF. 

### 3.2. Facilitator Training

The programme includes a two-day training session to train facilitators in the underpinning theories, the curriculum delivery and use of supporting resources. A guide on facilitation skills is also included on how to prevent and manage challenging interactions, such as one participant talking too much, being too shy to contribute, negative talk, not taking things seriously, going off on a tangent/racing ahead and what to do when a participant says they are an expert. Facilitators practice these skills during training and are encouraged to reflect after sessions. Facilitators were provided with a manual containing a curriculum of session plans for each session. Each session plan included the overall aim, learning outcomes, physical resources required and suggested online resources for sign-posting. Facilitator preparation guidance, the session plan and facilitator notes were also included. 

### 3.3. Supporting Resources

A number of colourful and engaging resources were created specifically for the PRE-STARt programme to ensure interactivity during the workshops. These included a colourful snakes and ladders activity mat plus oversize dice and counters, true/false cards and wipe-clean goal setting cards. There was a set of activity cards with 10-min physical activities (e.g., balloon badminton) which would be done at light intensity with sporadic moderate intensity bursts. Sessions also included activities that used easily sourced resources, such as local food packaging. 

### 3.4. Programme Curriculum

PRE-STARt sessions were designed to be delivered weekly by the same trained facilitator. Each 90-min workshop includes three sections: “Getting Started”, “Get Going” and “Let’s Go”. Each “Getting Started” section briefly recaps the key points from the previous week. Goals are reviewed by using a “snakes and ladders” game idea where the snakes are the challenges identified by the families in making the behaviour change and ladders are the facilitators. Within the “Get Going” (main), the new topic is introduced. The eight topics covered are presented in the graphical abstract. Within the “Let’s Go” section families are given an opportunity to set themselves a single goal based on the topic of the day or to continue working on a previous goal. Participants identify any potential barriers and facilitators (“snakes and ladders”) to achieving their goal. Families also engage in ten minutes of fun physical activity which they choose at random from the pack of activity cards. Where possible, standing or moving within the room was encouraged during the workshop to support the message of reducing sitting time and keeping active. 

### 3.5. Scope for Adaptations

PRE-STARt has the flexibility to allow adaptation for different cultures and needs. It can be delivered to families in a setting that maximises recruitment and retention of families and by a facilitator from any background, once they have been trained in the curriculum content and the underpinning theory and philosophy. The sessions are ordered to allow for building of messaged over the eight weeks. However, other than the first, last and the physical activity topic sessions, there is scope to alter the order. We have planned PRE-STARt session content, sections within session, length and dose (i.e., frequency, duration) to optimise chances of success. Any adaptation of these must be reported as per TIDieR checklist items [31].

## 4. Discussion

We have presented the development process of a family-based lifestyle programme that could be tested and implemented across five European sites. Documenting the intervention development phase ahead of a trial allows for the opening of “the black box” of the methods, processes and resulting unique decisions occurring during intervention development [24]. The outcome of the development process has been a programme for young people and their families ready for formal testing and a development process that is transparent and can add to the knowledge of how a programme was developed in five European sites.

There is an increasing need for effective and sustainable solutions to curb the growing T2D epidemic, in particular as T2D and obesity develop due to interactions between biological, behavioural, environmental and societal drivers [20]. Public health and community efforts aiming to tackle energy balance behaviours in order to affect obesity status are a popular panacea. Approaches must be agile to take on board the latest evidence and react to local clinical or public health need. While obesity-related programmes are more common in schools (83% of studies in a recent review [21]) our decision to design PRE-STARt primarily as a family-based programme is supported by the social ecological model [22]. Despite this, reviews found that school-based obesity programmes with direct parental involvement were limited, reporting was incomplete [19], but they were still more likely to be successful [18]. Direct parental involvement was defined as requesting parents to attend education sessions, family behaviour counselling or parent training sessions [19]. Across the literature, the extent of parental involvement or engagement varies and can be achieved through newsletters, homework, invites to family events without reporting of the level of engagement or uptake by parents, or, as in the landmark HEALTHY T2D prevention in schools trial [34], family challenges. 

Without doubt, multicomponent interventions will lead to more success [18]. If shown to be effective, we see PRE-STARt as one option for inclusion in “midstream” (individual behaviour change in specific settings such as the community or school) or “downstream” (health service) approaches [35] to T2D prevention or general healthy lifestyles promotion in young people and their families. For any approach to be a success, parents, schools and the after-school setting would work together to ensure that changes made in one setting are supported in the others in tandem. While the inclusion of wearable technology may be prohibitive for programmes with large numbers of participants, since this work was done, devices have lowered in prices and mobile phone apps have similar capabilities. Therefore, any device that allows participants to set and monitor goals that align with intervention programme goals can be used.

The MRC describe four phases for complex interventions of development, feasibility/piloting, evaluation and implementation. Only recently has a taxonomy around intervention development been produced [36]. We have described the development of the programme and the piloting of the sessions. The PRE-STARt development would align to the “target population-centered” and “theory- and-evidence-based” approaches [36]. Specific philosophies of how the programme should be run were included from the outset. Literature often does not report this and the philosophy might be taken for granted. PRE-STARt is theory-driven and we have outlined a number of underpinning behaviour change and learning theories.

There are a number of marked strengths to this development work. We have reported the process alongside two reporting checklists [25,31] to support replication. The written curriculum, resources and training manual have been culturally tailored, use up-to-date evidence and are underpinned by theory and philosophy, which will support standardised delivery within and between sites. 

PRE-STARt has the flexibility to allow adaptation for sites with different cultures and needs and to be delivered to families as part of a community, clinical or school prevention offering (e.g., a programme being funded by Public Health as part of a T2D prevention effort where families are invited to attend through schools with a certain socio-demographic or health need). Materials are easily translated to local languages and any site-specific changes were, and can be, made once they do not affect the underlying messaging, theory or philosophy. It is clear that family-based approaches improve chances of effectiveness [37] and PRE-STARt has been developed to be delivered to families. Future work may look at how and whether PRE-STARt can be delivered to young people alone (e.g., in schools), once the philosophy is followed, while involving families in other ways via schools or communities. The design of the PRE-STARt curriculum, resources and training allows sessions be delivered by a non-healthcare or an educational professional. Regardless of background, it is crucial that the facilitator be trained in the programme philosophy and also that any tailoring or modifications in delivery be reported. Our next steps will be a feasibility or pilot trial to gather data on recruitment and retention, as well as preliminary evidence of efficacy of behaviour change. The importance of the stakeholder contributions cannot be underestimated. Testing our resources and session and having the up-front stakeholder input provided valuable feedback on the session content, format and delivery. Key decisions on content such as taking the emphasis off T2D and more general healthy lifestyles came from stakeholders, as well as feedback on resources. Furthermore, a session around reducing sitting was included as sedentary behaviours are an important and, as stakeholders felt, novel behavioural target with few programmes measuring time spent sedentary as an outcome [17]. Although a substantial amount of resource and session testing with young people was done, the programme would have benefited from more feedback from a variety of audiences at all five European sites. In particular, work to identify who is best placed to facilitate programme delivery and whether and how the school is best placed to be involved.

Much stakeholder work was undertaken alongside the programme development in Table S1 in setting up for the main trial (e.g., identifying recruitment routes) and future implementation (e.g., identifying agencies who may deliver such a programme in the community or health service). There is a call to consider these decisions more upfront [36,38] in the planning stages of interventions. 

## 5. Conclusions

We have presented the development process of a family-based lifestyle programme designed specifically for T2D prevention in young people. Reporting on the intervention development process, which includes stakeholder input, could yield a flexible approach for use in this emerging ‘at risk’ group and their families.

## Figures and Tables

**Table 1:** PRE-STARt philosophy.

Philosophy	Details	Implications for Delivery
Families are ultimately responsible for the lifestyle choices they make.	Each family comes to the programme with their own set of beliefs, attitudes, behaviours, practices and household rules regarding physical activity, sedentary behaviour and healthy eating. It is widely acknowledged that the majority of the day-to-day decisions around physical activity, sedentary behaviour and healthy eating which will affect future health are made by the individual and their family. If individuals (families) are equipped with relevant information and appropriate skills they are able to make informed decisions for themselves (family) about making any changes in physical activity, sedentary behaviour and healthy eating.	Therefore, the facilitators were responsible for ensuring that individuals and families were provided with honest, up-to-date evidence-based information regarding healthy lifestyle choices. They were also responsible for ensuring individuals and families were supported to make their own action plan.
Families want to maximise their quality of life and will make decisions accordingly.	In general, individuals are motivated to maximise their quality of life and will make decisions accordingly. Quality of life will not always match the facilitators’ view. The aim of the workshop is to support everyone to make what they perceive to be the best decision for themselves and their family in order to progress to the best quality of life as they perceive it to be. This belief moves away from a paternalistic nature of healthcare and the notion that workshop facilitators know best.	Therefore, facilitators were responsible for ensuring that individuals and families were supported in processing and understanding the information provided to them and ensuring that everyone is treated nonjudgmentally and with respect regardless of how they decide to manage their lifestyle. Finally, they had to ensure that no-one was excluded from the group should they wish not to self-manage at any time, and in these cases they would be invited to participate in the future, as an individual’s readiness to be an active self-manager will vary over time.
The family is best placed to identify any barriers to implementing the new changes and identify any solutions to these.	In general, the majority of barriers to self-management are to be found in the individual’s personal and social world. Families can decide on what changes will work for them	Facilitators were responsible for ensuring warmth and empathy were demonstrated in all educational interactions, that everyone was given an opportunity to reflect on possible barriers to their self-management, that individuals were supported in developing general self-management skills such as goal setting, action planning and problem solving, and that individuals were supported in specific self-management skills, such as monitoring physical activity.

**Table 2:** COM-B (capability, opportunity, motivation behaviour) theoretical framework for the PRE-STARt programme.

COM-B	Theoretical Domains Framework	Behaviour Change Techniques	PRE-STARt Components
Psychological capability	Knowledge	Health consequences Feedback on behaviour	Information learning on moving more (session 2), healthy eating (session 3), sitting less (session 4), eating breakfast (session 5), healthy snacks (session 6), treats and fast food (session 7), myths and truths (session 8) Fun MVPA games, opportunity throughout all sessions for group discussion on what went well, what has not gone well.
	Skills	Graded tasks Behavioural practice Habit reversal Habit formation	Set easy-to perform tasks and group challenges to perform behaviour (i.e., design school breakfast club, place food on food map, exercise continuum, break sitting time during sessions). Discussions and participation in challenges that covered benefits of sitting, balanced diet and exercise. Prompted discussions on eating breakfast vs. skipping breakfast, sitting vs. breaking sitting, reading labels to review habit behaviour.
	Behavioural regulation	Self-monitoring of behaviour	Self-monitor of MVPA by providing activity monitors that gave data on step count, and sitting time.
Reflective motivation	Beliefs about capabilities	Focus on past success	Prompted group discussions in every session which promotes sharing experiences of having a go at making a lifestyle change and being successful; for example, building better breakfast, making better snack choices.
	Optimism	Verbal persuasion to boost self-efficacy	In every session, there was opportunity for individual self-reflection and small and large group discussion to consider making lifestyle change (however small) though setting small achievable goals, identifying personal barriers and solutions.
	Beliefs about consequences	Pros and cons	Generated pros and cons of behaviours (healthy eating, eating breakfast, fast foods), and weighed them up by discussing and by participating in challenges.
	Intentions	Commitment	Provided a foundation in session 1 for participants to commit to the programme, by introducing interactive activities and finishing sessions (2–8) with an action plan to revisit for next sessions.
	Goals	Goal setting (behaviour) Review of behaviour goals Action planning	In sessions 2–8, participants were provided with an action plan to set realistic and tangible goals. At the end of each session (2–7), behaviour goals were reviewed. Challenges provided the opportunity to discuss solutions to barriers and discuss next steps to behaviour change.
Social opportunity	Social influences	Social support Encouragement Modeling (demonstrating the behaviour)	Sessions were delivered in groups (children and parents). Group dynamic allowed for social support and encouragement amongst children and parents. Behaviours were modelled in the form of activities and challenges in each session to demonstrate positive behaviour. Suggestions and learning were drawn out of the group rather than being told or dictated by the facilitator providing opportunities for various learning. At the end of every session everyone engaged in fun achievable 10 min physical activity session to encourage children and families in physical activity games.

## Supplementary Materials

The following are available online at https://www.mdpi.com/2227-9067/8/2/69/s1, Table S1: Programme development activities undertaken, Table S2: UK site-led activities undertaken in each cycle, Table S3: Details of the UK site testing and feedback sessions, Table S4: TIDieR (template for intervention description and replication) checklist which identifies the information to include when describing an intervention and the location of the information.
