# Supplementary material for: Development of an Interactive Lifestyle Programme for Adolescents at Risk of Developing Type 2 Diabetes: PRE-STARt
Source: Children (Basel). 2021 Jan 21;8(2):69. doi: 10.3390/children8020069 (PMC7912284; doi:10.3390/children8020069)
Supplement: Supplementary file 1 [file children-08-00069-s001.pdf]

Table S1. Programme development activities undertaken

Number	Timeframe	Country	Event Type	Detail	Outcomes related to PRE-STARt
1	Q1	UK	Stakeholder meeting	Specific stakeholder held by LDC specifically for the PRE-STARt study	• Guidance on content for the intervention<br>• Discussions on how to communicate with parents/guardians
2	Q1	UK	Meetings with programme delivery groups	Meetings with University of Leicester outreach group (HERO), Leicester City Healthy Schools Network and Leicester Ramblers.	• Example materials and delivery approaches<br>• Better understanding of how to engage with young people
3	Q3	UK	School visit to LDC	Crown Hills Community College young leaders for health and Well-being visited the LDC and were given a presentation about the PRE-STARt study	• Better understanding of how to engage with young people<br>• Guidance on content for the intervention
4	Q3	UK	Nurse Network meeting	Presentation about the PRE-STARt study to 13 school nurses from across Leicester City	• Ideas generated for topics for the intervention workshops
5	Q3	UK	School Health Fair	Stand held delivering information about type 2 diabetes and shared some potential intervention activities	• Feedback received on potential intervention activities<br>• Generated ideas on types of topics which could be included in intervention workshops<br>• Provided experience of interacting with young people
6	Q4-Q5	Germany	Visit to local obesity outpatient clinic	Team visit to local obesity outpatients programmes for overweight and obese children and adolescents (KLAKS)	• Share ideas about the healthy lifestyle messages in the intervention
7	Q4	UK	Stakeholder meeting	Providing an update on the work so far and getting attendees to vote for and give feedback on key decisions	• eVoting system used to make decisions on six questions (the option in bold is the option the majority of the attendees chose)<br>(a) Where should the workshops take place?

Number	Timeframe	Country	Event Type	Detail	Outcomes related to PRE-STARt
					1. At a school<br>**2. In a community setting**<br>3. In a health centre<br>4. In a research centre<br>(b) What timetable should they follow?<br>**1. A few workshops in quick succession (i.e. once a week for 4 workshop) and then more spread out (i.e. once a month for 8 workshops)**<br>2. Once a month over the school year<br>3. Over the Summer<br>4. Once a week over the course of a school term<br>(c) What should the delivery be like?<br>1. One to one workshops (i.e. one family)<br>2. Splitting the parents and young people<br>3. All families in together for the whole workshop<br>**4. Families together and splitting when appropriate**<br>(d) **What length should each workshop be?**<br>**1. 90 minutes**<br>2. 3 hours<br>3. Depends on how many workshop there will be in total<br>4. Under an hour<br>(e) What style should the workshops be delivered in?<br>1. Allow participants access to paper resources and online content<br>2. Discussions<br>3. Practical tasks and fun games<br>**4. All of the above**<br>• Feedback on potential practical activities<br>• Ranking session topics from a list of 27 potential topics

Number	Timeframe	Country	Event Type	Detail	Outcomes related to PRE-STARt
					• Better understanding on communication strategies for parents/guardians
8	Q5	Spain	Stakeholder meetings	To inform the local stakeholders of the project	• Establish an active, informed stakeholder group
9	Q8	UK	CLAHRC East Midlands Young People Event	Workshop and Q&A session about the PRE-STARt study and intervention including sharing of intervention activities	• Feedback on intervention activities and study materials<br>• Young Leaders (see 08/07/2014) feedback about experience of taking part in research
10	Q9	UK	School visit to LDC	Secondary school students visited LDC to learn about research. A presentation on PRE-STARt activities was also delivered.	• Feedback on the appropriateness of intervention activities
11	Q11	Spain	Stakeholder meetings	To inform the local stakeholders of the status of the project	• Maintained an active, informed stakeholder group
12	Q13	Spain	Stakeholder meetings	To inform the local stakeholders of the status of the project	• Maintained an active, informed stakeholder group
13	Q13	UK	School, visit to LDC	Student health ambassadors from Kingswood Academy in Corby visited the LDC.	• Feedback on intervention activities
14	Q15	UK	School Health Fair	Autumn 2014 research fair	Autumn 2014 research fair plus<br>• Feedback on actual intervention activities

Table S2. UK site-led activities undertaken in each cycle

**Iterative Cycle One**	Working group established
	Scoping literature search
	Established theories and philosophy
	Broad workshop curriculum agreed
	First draft of workshops 3 and 6 and resources
	Broad curriculum and workshops 3 and 6 sent to collaborating countries for comment
	Feedback received via telephone and email
	Remaining workshops (1, 2, 4, 5, 7 and 8), resources and 10 min activity cards for 'Let's Go' section drafted
	Five testing and feedback conducted in the UK to gain feedback on the first draft of the workshops and resources *(see Table S3 for more detail)*

**Iterative Cycle Two**	Second draft of workshops were produced and resources adapted based on all feedback gathered throughout cycle one.
	All reviewed by Head of English teacher from a UK school to ensure consistency with teaching methods designed to support young people's learning. Feedback incorporated.
	All reviewed by Consultant Clinical Psychologist involved in establishing the theories and philosophy. Feedback incorporated.
	Site visit: Spain<br>Shared style of delivery and, content and resources for all workshops. Workshop 1 was re-designed based on feedback. Cultural adaptations were considered and integrated.
	Skype meeting: Portugal<br>Shared style of delivery and, content and resources for all workshops, documentation was emailed prior to the meeting for review. Feedback was provided both verbally during the call as well as written via tracked changes to documentation.
	Teleconference: Germany<br>Shared style of delivery and, content and resources for all workshops, documentation was emailed prior to the meeting for review. Feedback was provided both verbally during the call as well as written via tracked changes to documentation.
	Teleconference: Greece<br>Shared style of delivery and, content and resources for all workshops, documentation was emailed prior to the meeting for review. Feedback was provided both verbally during the call as well as written via tracked changes to documentation.
	Teleconferences: All sites<br>To look at specific queries raised by collaborating countries, in order to gain consensus of refinements to be made.
	Facilitator training for pilot delivery took place in the UK
	Pilot at inner city school in UK to test out and gain feedback on second draft of workshops and resources

**Iterative Cycle Three**	Final version of workshop curriculum and accompanying resources finalised.
	Final curriculum and resources shared with all collaborating countries (along with Theories and Philosophy document) for final considerations regarding specific cultural adaptations, as well as for translation.
	Cultural adaptations received, agreed and integrated. Translations of all documentation received. Final culturally adapted and translated versions of the curriculums distributed to collaborating sites.

Table S3. Details of the UK site testing and feedback sessions

First cycle					Second cycle
**Group 1**	**Group 2**	**Group 3**	**Group 4**	**Group 5**	**Group 1**
Where: Inner city school 1<br>With: 60 students<br>Objective: To test and gather feedback on the Activity Cards	Where: Inner city school 2<br>With: 5 students (2x 12 years, 14 years, 2x 15 years) and 1 parent (teacher)<br>Of which 2 mixed race, 4 boys, 1 girl.<br>Objective: To test and gather feedback on workshop curriculum and resources	Where: Inner city school 3<br>With: 11 students (aged 12-13) and 1 teacher, of which all South Asian, 7 boys, 4 girls.<br>Objective: To test and gather feedback on workshop curriculum and resources	Where: School on outskirts of city<br>With: 8 students (aged 13-14) and 1 teacher, of which 2 South Asian, 1 African Caribbean and 5 Caucasian British/European, 4 boys, 4 girls.<br>Objective: To test and gather feedback on workshop curriculum and resources	Where: Rural school<br>With: 5 students (aged 1x 12, 3 x 13, 1 x 15)<br>All white British, 4 girls, 1 boy.<br>Objective: To test and gather feedback on workshop curriculum and resources	Where: Inner city school 3<br>With: 9 students (4 girls (2 South Asian and 2 African Caribbean) & 5 boys (all South Asian) and 2 parents/guardians (1 male South Asian and 1 female African Caribbean).<br>Deprived inner city school where English is often second language.<br>Objective: To test out and gain feedback on second draft of workshops and resources and the self-monitoring device

Table S4. TIDieR (Template for Intervention Description and Replication) Checklist which identifies the information to include when describing an intervention and the location of the information

	Item	Page
	**BRIEF NAME**	
**1.**	Provide the name or a phrase that describes the intervention.	2,5
	**WHY**	
**2.**	Describe any rationale, theory, or goal of the elements essential to the intervention.	2,3
	**WHAT**	
**3.**	Materials: Describe any physical or informational materials used in the intervention, including those provided to participants or used in intervention delivery or in training of intervention providers. Provide information on where the materials can be accessed (e.g. online appendix, URL).	4,5,9
**4.**	Procedures: Describe each of the procedures, activities, and/or processes used in the intervention, including any enabling or support activities.	Figure2, Table 2
	**WHO PROVIDED**	
**5.**	For each category of intervention provider (e.g. psychologist, nursing assistant), describe their expertise, background and any specific training given.	9,11
	**HOW**	
**6.**	Describe the modes of delivery (e.g. face-to-face or by some other mechanism, such as internet or telephone) of the intervention and whether it was provided individually or in a group.	5
	**WHERE**	
**7.**	Describe the type(s) of location(s) where the intervention occurred, including any necessary infrastructure or relevant features.	11
	**WHEN and HOW MUCH**	
**8.**	Describe the number of times the intervention was delivered and over what period of time including the number of sessions, their schedule, and their duration, intensity or dose.	N/A
	**TAILORING**	
**9.**	If the intervention was planned to be personalised, titrated or adapted, then describe what, why, when, and how.	N/A
	**MODIFICATIONS**	
**10.ǂ**	If the intervention was modified during the course of the study, describe the changes (what, why, when, and how).	N/A
	**HOW WELL**	
**11.**	Planned: If intervention adherence or fidelity was assessed, describe how and by whom, and if any strategies were used to maintain or improve fidelity, describe them.	N/A
**12.ǂ**	Actual: If intervention adherence or fidelity was assessed, describe the extent to which the intervention was delivered as planned.	N/A

Note: N/A is used for an item which is not applicable for the intervention being described.
